# Life and Death in the Emotions

**Published:** 1897-04

**Authors:** 


### Life and Death in the Emotions.

Whatever can flush a face or pale it, whatever can bathe the body in hot perspiration or chill it with dampness, whatever can nerve one with strength or prostrate with weekness, whatever can cause rapture of blood vessels by pressure, or faintness from weakening circulation, must certainly have wonderful power and influence upon the standard of physicial condition which we call health. Instances are on record where a mother after being very angry has nursed her child, and it immediately went into convulsions and died, so great had been the effect of her emotions upon her milk. It is not uncommon for authors to suffer with extremely cold feet while at literary work. Many speakers, (as well as soldiers) are troubled with diarrhea, or with over activity of the kidneys, immediately preceding a public effort. Indeed, the variety of manifestations of this principle is almost as great as is the number of people engaged in pursuits in which the emotional nature is taxed or the emotions play an important part.

The writer was once an invalid and expected to die. His physicians said: "We can do nothing to help him because he knows his own condition so well that we can inspire no hope in him. When we sound his lungs he hears the dull thud or the light resonance and he knows what it means as well as we," and there never was an improvement until hope was inspired, and I am confident never could have been.

I have patients in my care to-day whose mournful state of mind over their condition is more difficult to overcome than their disease and more prostrating in its effects upon them.—C. A. Church, M.D., in *Health*.

